# Repetition and severity of suicide attempts across the life cycle: a comparison by age group between suicide victims and controls with severe depression

**DOI:** 10.1186/1471-244X-9-62

**Published:** 2009-09-29

**Authors:** Louise Brådvik, Mats Berglund

**Affiliations:** 1 Department of Clinical Sciences Lund, Division of Psychiatry, Lund University Hospital, Lund, Sweden; 2 Department of Clinical Alcohol Research, University Hospital MAS, Malmö, Lund University, Sweden

## Abstract

**Background:**

Suicide attempts have been shown to be less common in older age groups, with repeated attempts generally being more common in younger age groups and severe attempts in older age groups. Consistently, most studies have shown an increased suicide risk after attempts in older age. However, little is known about the predictive value of age on repeated and severe suicide attempts for accomplished suicide. The aim of the present study was to investigate the reduced incidence for initial, repeated, or severe suicide attempts with age in suicide victims and controls by gender.

**Methods:**

The records of 100 suicide victims and matched controls with severe depression admitted to the Department of Psychiatry, Lund University Hospital, Sweden between 1956 and 1969, were evaluated and the subjects were monitored up to 2006. The occurrence of suicide attempts (first, repeated, or severe, by age group) was analysed for suicide victims and controls, with gender taken into consideration.

**Results:**

There was a reduced risk for an initial suicide attempt by older age in females (suicide victims and controls) and male controls (but not suicide victims). The risk for repeated suicide attempts appeared to be reduced in the older age groups in female controls as compared to female suicide victims. The risk for severe suicide attempts seemed reduced in the older age groups in female suicide victims. This risk was also reduced in male controls and in male controls compared to male suicide victims.

**Conclusion:**

In the older age groups repeated attempts appeared to be predictive for suicide in women and severe attempts predictive in men.

## Background

Mood disorder is the individual diagnosis with the greatest impetus on suicide. Among completed suicides 29% to 88% (mean 52%) could be considered to have suffered from such a disorder [1], and there is an increased risk in severe depression as compared to major depression in general [2,3]. A suicide attempt is known to be one of the main predictors for suicide in depression [4-7].

Suicide attempts have consistently been found to be more common in younger age groups [8-13]. In general, repeated suicide attempts have been shown to be more common among young people [10,14] or in middle age [15,16]. By contrast, older people appear to make more severe suicide attempts [16-20]. Consistent with these findings, several studies have shown that increasing age at the time of the suicide attempt is a risk factor for accomplished suicide [21-30] By contrast, some investigators have found an increased risk for suicide in younger age groups by intoxication [31] or suicide in general [23,28]. A gender difference in suicide risk after attempt related to age has often been observed, but findings have been inconsistent. The risk has been shown to be increased in older age groups for women but not men [22,25,27] or men only [24,26,28]. By contrast, some investigators have found an increased risk for suicide after a suicide attempt with age for both men and women [23,29,30]. An increased risk for suicide for young suicide attempters has also been found, for men [23], women [28], or from intoxication for both sexes [31]. Finally, a recent study has shown that the overall gender ratios for deliberate self-harm conceal important changes in ratios across the life cycle [32].

To summarise, incidences of suicide attempt are known to be reduced in older age groups, and in particular the rate of repeated attempts is reduced. By contrast, the relative rate of severe suicide attempts has been shown to be greater in older age groups. Consistently, most studies found an increased suicide risk after attempts in older age.

However, to our knowledge, there has been no investigation into the predictive value of age at repeated and severe suicide attempt for accomplished suicide by gender.

The Department of Psychiatry in Lund, Sweden, has multiaxial ratings on all patients treated as inpatients during the time period from 1956 to 1969. There were 100 suicide victims with a primary severe depression at index admission. A blind record evaluation on suicide victims and matched controls has been performed, including non-fatal suicidal acts.

The aim of the present study was to compare the incidences of suicide attempt during the entire lifespan by age group in suicide victims and controls, with gender taken into consideration. The following questions were addressed: was there any reduction of incidence for initial, repeated, or severe suicide attempts with age in suicide victims or controls by gender, and was there any significant difference between suicide victims and controls by gender?

## Methods

### Sample

From 1949 to 1969, all inpatients at the Department of Psychiatry, University Hospital, Lund, Sweden, were rated on a multiaxial diagnostic schedule at discharge [33]. The diagnosis of severe depression/melancholia was introduced in 1956. Between 1956 and 1969 a total of 1,206 (506 men and 700 women) out of about 7,000 patients received this diagnosis. This database enabled the selection of patients with a prospectively rated severe depression/melancholia for an investigation into suicide. The very long-term follow-up (to 2006) enabled the collection of a fairly large number of accomplished suicides.

Their mortality was followed-up in three sessions: to 1 January 1984 [34], to 1 January 1998 [35] and for the present study to 1 May 2006. There were 116 suicide victims up to 2006. Out of these 103 had taken their life up to 1984, another 11 up to 1998, and 2 more up to 2006.

Deceased persons were grouped according to the primary cause of death as classified by the Swedish Central Bureau of Statistics using the International Classification of Disease (ICD) [36]. Undetermined suicides were excluded.

The case records were performed for a thorough evaluation of the suicide victims and matched controls from the total sample, in which the rater was unaware of the suicidal outcome [37], and in a similar procedure at second and third follow-up. By using a blinded procedure, we could avoid the usual bias inherent in retrospective evaluation. Secondary depressions were excluded according to research diagnostic criteria [38], mainly alcoholism. Thus we obtained data on 100 completed suicides, 44 men and 56 women, with primary severe depression. Matched controls, one for each suicide victim, were selected by diagnosis, sex, and age.

A retrospective diagnosis according to the Diagnostic and Statistical Manual of Mental Disorders, fourth edition (DSM-IV) [39] has been performed (by LB) based on the symptoms reported in the records to validate the diagnoses performed by the senior doctors at discharge. It was found that 91% of the patients met the criteria for major depressive disorder with melancholic (296.23) or psychotic features (296.24), when in a depressive phase. Though the case records were carefully written and very informative, individual symptoms might have been under-reported. Thus the actual number was probably higher. In the suicide group 20 patients had experienced at least 1 episode of elevated mood at some point, indicating bipolarity, versus 20 in the control group. There were 56 suicide victims and 55 controls who at some time had experienced an episode of psychotic depression.

### Record evaluation

The entire course of depression up to the death of the suicide victims and a corresponding date for the matched controls was studied. There were a total of 1,505 observation years in the suicide group and 1,531 in the control group. In the suicide group 60 patients (25 men and 35 women) were reported to have made 133 suicide attempts and in the control group 34 patients (17 men and 17 women) were reported to have made 76 suicide attempts.

The occurrence of suicide attempt was related to age groups and number of observation years for suicide victims and controls. A majority of the suicide attempts were made in close connection to admission (often a cause of admission), 89% of the suicide attempts in the suicide group and 84% in the control group. In all but three cases (one male suicide victim, one female suicide victim and one female control) the suicide attempt was reported to be 'recent', which is why the age at suicide attempt appears certain for all but three cases.

The proportion of repeated and severe attempts by gender is presented in Table 1. The results in this table are an extension of a previous study [7] including 11 more suicide victims (as the findings are similar we present them under 'Methods'). Suicide victims make more attempts than controls and women repeat attempts more often than men and also more often make severe attempts. Repetition and severity do not appear to discriminate between suicide victims and controls for either gender.

**Table 1:** Number of suicide attempts for suicides and controls by gender

	**Females**		**Males**	
	
	**Suicide victims, n = 56**	**Controls, n = 56**	**Suicide victims, n = 44**	**Controls, n = 44**
No. of suicide attempters	35 (63%)^a^	17 (30%)	25 (57%)	17 (39%)
No. of suicide attempts	93	51	40	25
Average no. of suicide attempts	1.66	0.91	1.1	0.57
No. of repeaters	19 (54%)	8 (47%)	8 (32%)	6 (35%)
No. of repeated suicide attempts	58	34	15	8
Average no. of attempts among repeaters	4.05	5.25	2.88	2.33
No. of severe attempts among attempters	13 (37%)	6 (35%)	6 (24%)	3 (18%)

^a^χ^2 ^= 11.63, *P *< 0.001.

Suicide attempt was scored according to Motto [40] and Weisman and Worden [41] and graded for severity in a previous study on these severely depressed patients [7]. The evaluation was based on the following definitions.

Suicidal gestures: an act of self-harm with little or no physical injury where the intent to die is not clearly stated.

Ambivalent suicide attempt: a patient initiates a suicidal act, which is potentially fatal, but interrupts this action and thus does not cause a great deal of self-damage.

Definite suicide attempt: life-threatening behaviour with a moderately high risk of death and low chance of rescue.

Severe suicide attempt: highly lethal suicide attempts, such as those requiring intensive care. Precautions against discovery and strong regret at failure to die are considered psychologically severe.

In the present study only severe versus non-severe suicide attempts were analysed.

The age at suicide attempt by order and severity was compared for suicide victims and controls. Violent methods of suicide attempt have not been shown to increase or decrease with age in a previous study and this factor is therefore not taken into account [42].

The study was approved by Lund University Medical Ethics Committee, 1985 and 2003.

### Statistics

A Poisson regression was used for comparisons between the age groups for 5-year intervals for suicide victims and controls by gender and between suicides and controls by gender (Stata/SE v. 9.2 for Unix; Stata, College Station, TX, USA). Two-tailed tests were used and the significance level was set at 5%.

## Results

### First suicide attempt

The incidences of initial suicide attempt were reduced with age for female suicide victims (Poisson regression, *P *< 0.041) and controls (Poisson regression, *P *< 0.022) and for male controls (Poisson regression, *P *< 0.020). However, there was no reduction in rates by age in the male suicide group.

### Repeated suicide attempts

**Figure 1:**
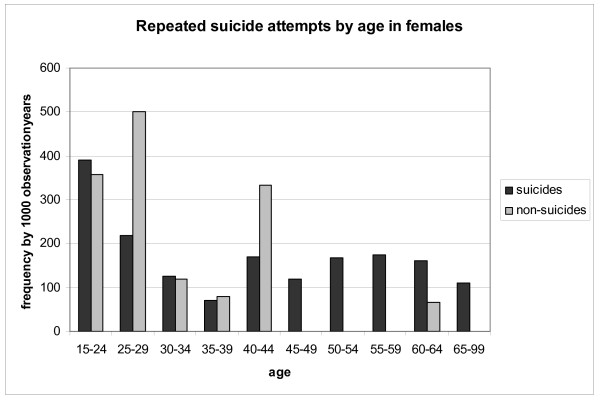
**Repeated suicide attempts by age in females**.

**Figure 2:**
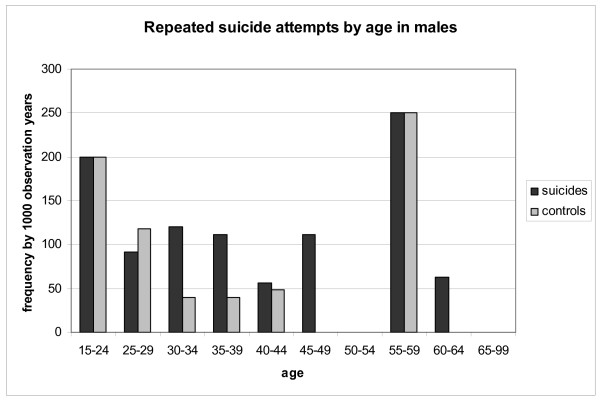
**Repeated suicide attempts by age in males**.

The distributions of repeated attempts by gender are presented in Figures 1 and 2. The rates of repetition were only reduced in the female control group (Poisson regression, *P *< 0.004). Female controls showed a significantly more reduced incidence of repetition with age when compared with female suicide victims (Poisson regression, *P *< 0.024).

This difference for reduced rates of repeated suicide attempt by age was not found in the male group.

### Severity of suicide attempts

The rate of severe suicide attempts was reduced in older age in the female suicide group (Poisson regression, *P *< 0.007). No such trend for reduction was found in the female control group. There was no significant difference between female suicide victims and controls.

In contrast, in the male group controls showed a reduced incidence of severe suicide attempts with older age (Poisson regression, *P *< 0.001). The incidence of severe attempts was also significantly more reduced in male controls as compared with male suicide victims (Poisson regression, *P *< 0.007).

## Discussion

### Main findings

First, the reduced incidences of suicide attempt in the older age groups for repetition and severity in suicide victims and controls were calculated. There were significantly reduced rates of first suicide attempt by age in all groups apart from male suicide victims. Decreased incidence of repeated attempts was shown to be significant for female controls only. Significantly reduced rates of severe attempt were shown for female suicide victims and male controls. Repetition and severity of suicide attempts, including accomplished suicide, by age group and gender are presented in Table 2.

**Table 2:** Repetition and severity of suicide attempts, including accomplished suicide, by age group and gender.

	**male controls**		**male suicides**			**female controls**		**female suicides**		
	
	**first**	**repeated**	**first**	**repeated**	**suicide**	**first**	**repeated**	**first**	**repeated**	**suicide**
15-24	2	1	5	3 (2)	2	4	5	8 (2)	9	2
25-29	5	2	4 (1)	2 (1)	2	2	8	1	7 (3)	1
30-34	1	1	2	3	3	1	3 (1)	2 (1)	4 (1)	2
35-39	1	1	1 (1)	2 (1)	2	3	3 (2)	3 (1)	3	2
40-44	2	1	1	1	6	1	14 (2)	4 (1)	8 (3)	4
45-49	1	0	2 (1)	1	4	1	0	3	6	4
50-54	1	0	4 (1)	0	4	1	0	6 (2)	8 (1)	5
55-59	2	2	0	2 (1)	4	3 (1)	0	3 (1)	7 (1)	9
60-64	2	0	4	1	3	1	1 (1)	3 (1)	4 (1)	9
65-99	0	0	2	0	14	0	0	2	2	7

Suicide attempts have been shown to be less common in older age groups as compared to younger ones in previous studies [8-10]. Furthermore, the rates of repeated attempts have been shown to be reduced with age [10]. Severe suicide attempts, by contrast, have shown increased rates in older age groups [17-20].

However, those studies did not differentiate between future suicide victims and controls, when repetition and severity of suicide attempts were taken into account. Female and male controls appear to become less suicidal with age. In the female group repetition is reduced and in the male group the severe attempt rates are reduced.

Second, we compared the reduced risk for repeated and severe suicide attempts during the life cycle between suicide victims and controls by gender. In the female group, controls showed significantly more reduced rates of repeated attempts as compared to suicide victims. In the male group controls showed significantly more reduced rates of severe attempts as compared to suicide victims.

The risk for suicide after a suicide attempt in older age groups has been found to increase for women according to some studies [22,23,27], but not according to others that showed an increased risk with age for men only [24,26,28]. The discrepancy between these findings might to some extent be due to the fact that repetition and severity has not been taken into consideration.

### Suicidal behaviour in suicide victims and controls by gender

Suicide attempts throughout the life cycle could be described as follows: (1) female suicide victims showed reduced rates of first suicide attempts and severe suicide attempts throughout their life course. However, they showed no reduced rates of repetition in the older age groups. (2) Female controls showed reduced rates of first suicide attempts and repeated suicide attempts in middle age, but no reduction of severe attempts. (3) Male suicide victims showed no pattern of reduction in risk for a first, repeated, or severe attempt in older age. (4) Male controls showed a reduced risk for a first and for severe attempts in older age.

Men and women showed different patterns of suicide attempt in older age groups, both confirming continuous suicidality in older age groups by severity or repetition, respectively, in future suicide victims.

Men who later accomplished suicide made more severe attempts later in life, as compared to controls. This finding is in agreement with the literature on severe suicide attempts in older age groups [17-20]. In the present study repetition of suicide attempts was more common in women than in men in future suicide victims as well as controls for all categories. This is in agreement with some previous studies [43,44] but contradictory to other studies, where men showed similar or somewhat higher rates of repetition than women [45,46]. The discrepancy may be due to the present sample consisting of severely depressed patients. Higher rates of repetition in older age groups have to our knowledge not been shown before. However, the comparison between female suicide victims and controls by repetition has not been made either. The continuous repetition in female suicide victims is noteworthy and repeated suicide attempt in older females should be taken seriously.

Finally, the facts that female controls make few repeated attempts after middle age and survive severe suicide attempts in older age groups are worth further exploration. So is the fact that male controls show reduced rates of severe attempts in older age groups. The mechanisms behind these findings may give clues on how to interrupt the suicidal process.

### Strengths and limitations

The present study was based on a sample of 1,206 patients with severe depression/melancholia, who had been rated on a multiaxial schedule and monitored for 37 to 50 years after their first admission with this diagnosis. The number of completed suicides was fairly high at 100. The agreement of diagnostics with DSM-IV appeared to be high, with at least 91% fulfilling the diagnostic criteria for major depressive disorder with melancholic or psychotic features. Only primary depressions were included, while depressions secondary to other disorders (mainly alcoholism) were excluded. As no depression was secondary to alcohol abuse, the impact of such abuse was diminished. The fact that the sample constitutes patients with a severe depression makes it less representative for a general suicide sample. However, these patients are at a particular high risk for suicide and are therefore worth studying.

We used a rather broad definition of self-harm, as intent is difficult to decide based on case records. The study started in 1984 and the same definitions were used in the two follow-ups in 1998 and 2006. Some more recent investigators also use a broad definition of self-harm without considering the degree of intent [47-49], which would include suicidal gestures and probably some aborted attempts (here considered ambivalent attempts). The latter have been described by Marzuk *et al*. [50] and been associated with actual suicide attempts [51]. Severe suicide attempts are similar to serious attempts as defined by Motto [40] as well as Beautrais [52] that is a need for intensive care after intoxication. However, in the present study, in contrast to Beautrais, suicide attempt by hanging and other violent methods were considered severe only if it caused damage (for instance asphyxia). This means that there is a more narrow definition for severe attempt by violent methods in the present study. High intent as suggested by Suokas and coworkers [53] as well as Weissman and Worden [41] was also considered severe.

Suicide attempt was evaluated against reports in the case records, as there were no personal interviews. Thus, the severity of the intent may be difficult to evaluate because of limited information.

Nevertheless, the suicide attempts have been continuously registered by case record evaluation, thus minimising the recall bias inherent in interviews later in life. However, there is always a risk that some suicidal behaviour is never reported if there is no need for medical intervention and thus missed out in a case record. The crucial point is whether report of repetition and severity is equally reliable for future suicides and controls. This could be assumed, but not proven.

Time of the suicide attempt and thus age at the event could be stated with certainty in 84% of the controls and 89% of the suicide victims, as the attempts were made in connection with hospitalisation or outpatient admissions. In only three cases were the suicide attempts reported to have occurred in the past, and in these cases there could possibly be recall bias. The remaining cases were recent at the time of contact to within a few months or so.

## Conclusion

The present study investigates the reduced incidence of suicide attempts throughout the lifespan concerning repetition and severity in suicide victims and controls by gender.

Repeated suicide attempts in women and severe attempts in men in older age appear to be risk factors for future suicide. The reason as to why female controls stop making repeated suicide attempts with age, and why they survive severe suicide attempts in older age, is worth further exploration. Another topic for future research is the reason why male controls do not make severe attempts in older age. Such reasons may give valuable information for the prevention of suicide.

## Competing interests

The authors declare that they have no competing interests.

## Authors' contributions

LB initiated the study, contributed to the design and drafted the manuscript. MB contributed to the design. Both authors read the manuscript.

## Pre-publication history

The pre-publication history for this paper can be accessed here:



## References

[B1] Lönnqvist JK, Hawton K, Van Heeringen K (2000). Psychiatric aspects of suicidal behaviour: depression. The International Handbook of Suicide and Attempted Suicide.

[B2] Helgason T (1964). Epidemiology of mental disorders in Iceland. A psychiatric and demographic investigation of 5395 Icelanders. Acta Psychiatr Scand.

[B3] Brådvik L, Mattisson C, Bogren M, Nettelbladt P (2008). Long-term suicide risk in depression in the Lundby cohort 1947-1997 - severity and gender. Acta Psychiatr Scand.

[B4] Sainsbury P, Roy A (1986). Depression, suicide and suicide prevention. Suicide.

[B5] Goldstein RB, Black DW, Nasrallah A, Winokur G (1991). The prediction of suicide. Sensitivity, specificity, and predictive value of a multivariate model applied to suicide among 1906 patients with affective disorders. Arch Gen Psychiatry.

[B6] Nordström P, Åsberg M, Åberg-Wistedt A, Nordin C (1995). Attempted suicide predicts suicide risk in mood disorders. Acta Psychiatr Scand.

[B7] Brådvik L, Berglund M (2002). Aspects of the suicidal career in severe depression. A comparison between attempts in suicides and controls. Arch Suicide Res.

[B8] Platt S, Bille-Brahe U, Kerkhof A, Schmidtke A, Bjerke T, Crepet P, De Leo D, Haring C, Lonnqvist J, Michel K, Philippe A, Pommereau X, Querejeta I, Salander-Renberg E, Temesvary B, Wasserman D, Sampaio Faria J (1992). Parasuicide in Europe: the WHO/EURO multicentre study on parasuicide. I. Introduction and preliminary analysis for 1989. Acta Psychiatr Scand.

[B9] De Leo D, Padoani W, Scocco P, Lie D, Bille-Brahe U, Arensman E, Hjelmeland H, Crepet P, Haring C, Hawton K, Lonnqvist J, Michel K, Pommereau X, Querejeta I, Phillipe J, Salander-Renberg E, Schmidtke A, Fricke S, Weinacker B, Tamesvary B, Wasserman D, Faria S (2001). Attempted and completed suicide in older subjects: results from the WHO/EURO multicentre study of suicidal behaviour. Int J Geriatr Psychiatry.

[B10] Corcoran P, Keeley HS, O'Sullivan M, Perry IJ (2004). The incidence and repetition of attempted suicide in Ireland. Eur J Public Health.

[B11] Schmidtke A, Bille-Brahe U, DeLeo D, Kerkhof A, Bjerke T, Crepet P, Haring C, Hawton K, Lönnqvist J, Michel K, Pommereau X, Querejeta I, Phillipe I, Salander-Renberg E, Temesváry B, Wasserman D, Fricke S, Weinacker B, Sampaio-Faria JG (1996). Attempted suicide in Europe: rates, trends and sociodemographic characteristics of suicide attempters during the period 1989-1992. Results of the WHO/EURO Multicentre Study on Parasuicide. Acta Psychiatr Scand.

[B12] Bernal M, Haro JM, Bernert S, Brugha T, de Graaf R, Bruffaerts R, Lépine JP, de Girolamo G, Vilagut G, Gasquet I, Torres JV, Kovess V, Heider D, Neeleman J, Kessler R, Alonso J, ESEMED/MHEDEA Investigators (2007). Risk factors for suicidality in Europe: results from the ESEMED study. J Affect Disord.

[B13] Kessler RC, Borges G, Walters EE (1999). Prevalence of and risk factors for lifetime suicide attempts in the National Comorbidity Survey. Arch Gen Psychiatry.

[B14] Sakinofsky I, Roberts RS (1990). Why parasuicides repeat despite problem resolution. Br J Psychiatry.

[B15] Kreitman N, Foster J (1991). The construction and selection of predictive scales, with special reference to parasuicide. Br J Psychiatry.

[B16] Hjelmeland H, Stiles TC, Bille-Brahe U, Ostamo A, Salander Renberg E, Wasserman D (1998). Parasuicide: The value of future suicidal intent and various motives as predictors of future suicidal behaviour. Arch Suicide Res.

[B17] Draper B (1996). Attempted suicide in old age. Int J Geriatr Psychiatry.

[B18] Hawton K, Harriss L (2006). Deliberate self-harm in people aged 60 years and over: characteristics and outcome of a 20-year cohort. Int J Geriatr Psychiatry.

[B19] Hepple J, Quinton C (1997). One hundred cases of attempted suicide in the elderly. Br J Psychiatry.

[B20] Merrill J, Owens J (1990). Age and attempted suicide. Acta Psychiatr Scand.

[B21] Holley HL, Fick G, Love EJ (1998). Suicide following an inpatient hospitalization for a suicide attempt: a Canadian follow-up study. Soc Psychiatry Psychiatr Epidemiol.

[B22] Nordentoft M, Breum L, Munck LK, Nordestgaard AG, Hunding A, Laursen Bjaeldager PA (1993). High mortality by natural and unnatural causes: a 10 year follow up study of patients admitted to a poisoning treatment centre after suicide attempts. BMJ.

[B23] Nordström P, Samuelsson M, Åsberg M (1995). Survival analysis of suicide risk after attempted suicide. Acta Psychiatr Scand.

[B24] Rygnestad T (1997). Mortality after deliberate self-poisoning. A prospective follow-up study of 587 persons observed for 5279 person years: risk factors and causes of death. Soc Psychiatry Psychiatr Epidemiol.

[B25] Skogman K, Alsen M, Öjehagen A (2004). Sex differences in risk factors for suicide after attempted suicide - a follow-up study of 1052 suicide attempters. Soc Psychiatry Psychiatr Epidemiol.

[B26] Suokas J, Lönnqvist J (1991). Outcome of attempted suicide and psychiatric consultation: risk factors and suicide mortality during a five-year follow-up. Acta Psychiatr Scand.

[B27] Hawton K, Fagg J (1988). Suicide, and other causes of death, following attempted suicide. Br J Psychiatry.

[B28] Iribarren C, Sidney S, Jacobs DR, Weisner C (2000). Hospitalization for suicide attempt and completed suicide: epidemiological features in a managed care population. Soc Psychiatry Psychiatr Epidemiol.

[B29] Nimeus A, Alsen M, Träskman-Bendz L (2000). The suicide assessment scale: an instrument assessing suicide risk of suicide attempters. Eur Psychiatry.

[B30] Reith DM, Whyte I, Carter G, MCPherson M, Carter N (2004). Risk factors for suicide and other deaths following hospital treated self-poisoning in Australia. Aust N Z J Psychiatry.

[B31] Buckley NA, Dawson AH, Whyte IM, Hazell P, Meza A, Britt H (1996). An analysis of age and gender influences on the relative risk for suicide and psychotropic drug self-poisoning. Acta Psychiatr Scand.

[B32] Hawton K, Harriss L (2008). The changing gender ratio in occurrence of deliberate self-harm across the lifecycle. Crisis.

[B33] Essen-Möller E, Wohlfahrt S (1947). Suggestions for the amendment of the official Swedish classification of mental disorder. Acta Psychiatr Scand.

[B34] Berglund M, Nilsson K (1987). Mortality in severe depression. A prospective study including 103 suicides. Acta Psychiatr Scand.

[B35] Brådvik L, Berglund M (2001). Late mortality in severe depression. Acta Psychiatr Scand.

[B36] Sweden's National Board of Health and Social Welfare (1987). Klassifikation av sjukdomar 1987. Systematisk förteckning (Swedish Version of the International Classification of Diseases, Ninth Revision (ICD-9)).

[B37] Brådvik L, Berglund M (1993). Risk factors for suicide in melancholia. A case record evaluation of 89 suicides and their controls. Acta Psychiatr Scand.

[B38] Spitzer R, Endicott J, Robins E (1978). Research diagnostic criteria. Arch Gen Psychiatry.

[B39] American Psychiatric Association (1994). Diagnostic and Statistical Manual of Mental Disorders.

[B40] Motto JA (1965). Suicide attempts. A longitudinal view. Arch Gen Psychiatry.

[B41] Weisman AD, Worden JW (1972). Risk-rescue rating in suicide attempt. Arch Gen Psychiatry.

[B42] Brådvik L (2007). Violent and non-violent method in suicide. Different patterns may be found in men and women with severe depression. Arch Suicide Res.

[B43] Runeson BS, Beskow J, Waern M (1996). The suicidal process in suicides among young people. Acta Psychiatr Scand.

[B44] Gibb SJ, Beautrais AL, Fergusson DM (2005). Mortality and further suicidal behaviour after an index suicide attempt: a 10-year study. Aust N Z J Psychiatry.

[B45] Kreitman N, Casey P (1988). Repetition of parasuicide: an epidemiological and clinical study. Br J Psychiatry.

[B46] Canetto SS, Sakinofsky I (1998). The gender paradox in suicide. Suicide Life Threat Behav.

[B47] O'Carroll PW, Berman AL, Maris RW, Moscicki EK, Tanney BL, Silverman MM (1996). Beyond the Tower of Babel: a nomenclature for suicidology. Suicide Life Threat Behav.

[B48] Hawton K, Fagg J, Simkin S, Bale E, Bond A (1997). Trends in deliberate self-harm in Oxford, 1985-1995. Implications for clinical services and the prevention of suicide. Br J Psychiatry.

[B49] Hawton K, Harriss L, Hall S, Simkin S, Bale E, Bond A (2003). Deliberate self-harm in Oxford, 1990-2000: a time of change in patient characteristics. Psychol Med.

[B50] Marzuk PM, Tardiff K, Leon AC, Portera L, Weiner C (1997). The prevalence of aborted suicide attempts among psychiatric in-patients. Acta Psych Scand.

[B51] Barber ME, Marzuk PM, Leon AC, Portera L (1998). Aborted suicide attempts: a new classification of suicidal behavior. Am J Psychiatry.

[B52] Beautrais AL (2003). Suicide and serious suicide attempts in youth: a multiple-group comparison study. Am J Psychiatry.

[B53] Suokas J, Suominen K, Isometsä E, Ostamo A, Lönnqvist J (2001). Long-term risk factors for suicide mortality after attempted suicide--findings of a 14-year follow-up study. Acta Psychiatr Scand.

